# Welfare of dairy cows in Kosovo and intervention thresholds for selected welfare indicators as suggested by farmers and veterinarians – ERRATUM

**DOI:** 10.1017/awf.2023.15

**Published:** 2023-02-15

**Authors:** E Zhitia, C Leeb, S Muji, C Winckler

*A non-unique DOI was erroneously assigned to this article at the time of its publication. The article has now been assigned a new DOI. Both the PDF and XML versions have been updated accordingly. The original and new DOIs of this article are provided below.*

*Original DOI:* 10.7120/09627286.31.4.006

*New DOI:* 10.1017/S0962728600032474
